# Breaking barriers: broadening neuroscience education via cloud platforms and course-based undergraduate research

**DOI:** 10.3389/fninf.2025.1608900

**Published:** 2025-07-16

**Authors:** Franco Delogu, Chantol Aspinall, Kimberly Ray, Anibal Solon Heinsfeld, Conner Victory, Franco Pestilli

**Affiliations:** ^1^Department of Humanities, Social Sciences and Communication, Lawrence Technological University, Southfield, MI, United States; ^2^Department of Psychology, The University of Texas at Austin, Austin, TX, United States; ^3^Department of Psychology, Oakland University, Rochester, NY, United States

**Keywords:** CURE, brainlife.io, sMRI, human brain volumetry, project based active learning, human brain parcellation, open-access neuroscience databases, broadening participation in computing

## Abstract

This study demonstrates the effectiveness of integrating cloud computing platforms with Course-based Undergraduate Research Experiences (CUREs) to broaden access to neuroscience education. Over four consecutive spring semesters (2021–2024), a total of 42 undergraduate students at Lawrence Technological University participated in computational neuroscience CUREs using brainlife.io, a cloud-computing platform. Students conducted anatomical and functional brain imaging analyses on openly available datasets, testing original hypotheses about brain structure variations. The program evolved from initial data processing to hypothesis-driven research exploring the influence of age, gender, and pathology on brain structures. By combining open science and big data within a user-friendly cloud environment, the CURE model provided hands-on, problem-based learning to students with limited prior knowledge. This approach addressed key limitations of traditional undergraduate research experiences, including scalability, early exposure, and inclusivity. Students consistently worked with MRI datasets, focusing on volumetric analysis of brain structures, and developed scientific communication skills by presenting findings at annual research days. The success of this program demonstrates its potential to democratize neuroscience education, enabling advanced research without extensive laboratory facilities or prior experience, and promoting original undergraduate research using real-world datasets.

## Introduction

Neuroscience remains one of the least inclusive STEM disciplines. Recent data shows that only 12% of neuroscience program applicants were from ethnic minorities, compared to 18% for medical schools and 23% for graduate schools overall (Abiodun, 2019). This scarcity of underrepresented minority (URM) students in neuroscience stems from several barriers. Historically, access to neuroscience data has been limited to the institutions generating it, often due to fears of “parasitic” practices, data misinterpretation by other researchers (Longo and Drazen, 2016), data governance challenges related to managing and sharing neuroimaging data in compliance with national and supranational regulations, as well as ethical constraints where participant consent may not allow sharing. Cost presents a major barrier for both students and institutions. Most neuroscience programs are housed in larger, PhD granting institutions, which tend to be more expensive than bachelor’s level institutions. These programs demand expensive technical equipment and infrastructure, which restricts adequate neuroscience training to larger, better-funded institutions. This limitation disproportionately affects underrepresented minority (URM) students, particularly in countries like the United States, where high tuition costs create significant financial barriers.^1^

The imbalance extends to faculty and researchers (Wheaton, 2021), resulting in fewer URM role models and mentors, further discouraging promising URM students from pursuing neuroscience. To diversify neuroscience and STEM fields, approaches must focus on extending research opportunities to resource-limited settings, particularly those serving URMs. Open science presents one such possibility to address these challenges.

The Open Science movement is not new to research, but its adoption in fields like human neuroscience where personal health information is often involved has been more complex than in others. Neuroscience data (e.g., MRI, EEG, intracranial recordings) is often rich in information that could potentially be used to re-identify individuals or reveal sensitive personal attributes related to mental health and cognitive abilities. This inherent sensitivity necessitates rigorous de-identification procedures, secure data storage, and carefully controlled access, all of which complicate and can slow down the broad adoption of open data practices compared to fields like astronomy or certain areas of physics, where data is less directly tied to individual human identity and privacy. While fields like genomics also face severe privacy constraints, neuroscience has its own unique set of challenges due to the direct link between the data and an individual’s cognitive and mental state, making the “mental privacy” aspect particularly salient (Jwa and Poldrack, 2022).

Open science refers to a set of practices encompassing open access publication, open research funding, open education, open data and materials sharing, open infrastructures (e.g., digital laboratories and libraries), open software tools, and the minimization of restrictive intellectual property rights (Gold et al., 2019; Vicente-Saez and Martinez-Fuentes, 2018). The goals of open science are to reduce research costs for all, promote data reuse to increase reproducibility, decrease scientific redundancy, invite in a larger diversity of people, and increase the rate of discovery and innovation (Gold, 2016; McKiernan et al., 2016; Ali-Khan et al., 2018). Sharing research assets lowers entry barriers for scientific participation significantly, bringing about a more level playing field for underrepresented groups and minority-serving institutions. Compared to the traditional, Western view of science as being about individual effort and competition, the language and ethos of open science reflects more prosocial, communal, and interdependent concepts, which in turn may be more attractive to people of color, women, and people from lower socioeconomic backgrounds (Murphy et al., 2020). Thus, open science can create a positive feedback loop in which greater inclusion fosters a culture that in turn welcomes greater inclusivity. To increase diversity in STEM, Murphy et al. (2020) call for “initiatives to allow for establishing education networks, training, resources, and data sharing,” along with “the establishment of cloud-based platforms and associated user communities for research asset sharing.” In other words, open education.

Open education focuses on the benefits of open science to decrease inequality in learning and to support lifelong learning (Blessinger and Bliss, 2016). Open education refers to the sharing of resources and assets that users are able to “retain, reuse, remix, and redistribute” (Wiley et al., 2014). Though the application of open science to education has received less attention overall compared to research (Heck et al., 2020), open neuroscience (Nielsen, 2020) and large-scale data sharing projects (van Essen et al., 2013; Gorgolewski et al., 2017) have strong potential to increase accessibility and diversity in neuroscience education and training (Markiewicz et al., 2021; Rokem et al., 2021; Milham et al., 2018), and several such efforts are underway. For example, the OpenNeuro program, evolving from previous initiatives in neuroscience data sharing, currently offers more than 1,400 datasets, including data from more than 60,000 participants, comprising multiple species and measurement modalities (Markiewicz et al., 2021). Similarly sized, the Adolescent Brain Cognitive Development (ABCD) study longitudinally tracks and openly shares the biological and behavioral development data of more than 10,000 children at 21 sites (Casey et al., 2018). Neurohackademy (NHD) students spend 2 weeks at the eScience Institute of the University of Washington learning how to analyze human neuroscience data and make the results shareable and reproducible. The online format of the 3-week, computational neuroscience summer school by Neuromatch Academy makes it more affordable for multilingual and multinational trainees around the world (van Viegen et al., 2021; Kording, 2021). Brainhack (BH) offers a novel, less formal workshop format with participant-generated content catering to the rapidly growing open neuroscience community. Brainhack includes hackathons, “unconferences,” and educational sessions with the goal of fostering new ideas, projects, and collaborations (Gau et al., 2021).

But while these provide useful models, they are limited with regard to the goals of inclusion, as they primarily focus on increasing the existing technical and computational skills of users, i.e., highly-skilled, well-educated trainees (graduate students, postdocs) and faculty with the aim of growing a data science-ready generation of Psychological and Brain Science Ph. D.’s. In short, these programs have been developed to facilitate access to complex neuroscience, advanced coding skills and data accessibility, and not necessarily to lower barriers of entry to neuroscience as a whole. To our knowledge, no existing open science/big data neuroscience educational program specifically targets undergraduate students. Thus, there is a pressing need to develop a curriculum with similar methodologies for content delivery as Brainhack, Neurohackademy, Advanced Computational Neuroscience Network, and Neuromatch Academy, but that both targets younger students with limited or no neuroscience or data science literacy, and is attractive to URMs and URM-serving schools.

A second limitation of existing big data (and other) training programs within neuroscience is a tendency toward a focus on *within-discipline* methods and skills. But, in truth, neuroscience is a multidisciplinary field where its foundations are built on biology, physics, and other STEM fields. To truly solve the diversity and accessibility problem within neuroscience, training needs to integrate expertise from neuroscience, computer science, engineering, the medical sciences, and high performance computing. One example of a big data training program that integrates these disciplines is the Advanced Computational Neuroscience Network (ACNN). ACNN workshops build *transdisciplinary* educational opportunities and interactive learning abilities as they promote the sharing of data management tools, online documentation, and training manuals. As of today, there is not a comparable resource for undergraduate students. One of the main goals of our study is to promote notions of multidisciplinarity and flexibility in undergraduate neuroscience education, in contrast with ultra specialized training in one technique, modality, hypotheses or brain area.

We describe here a pilot learning experience that provides an introduction in computational neuroscience to undergraduate students at Lawrence technological University, a primary undergraduate institution (PUI) located in the Metro Detroit area. The experience integrates open science and big data via a user-friendly cloud-computing virtual environment (brainlife), with course-based research experience model (CURE) with the goal of providing hands-on, problem-based learning in data neuroscience to undergraduate students with very limited or no previous knowledge in neuroscience.

Brainlife.io is a big data neuroscience cloud platform that brings together scientists, educators, and trainees from psychology, neuroscience, engineering, and computer science in order to lower barriers to entry and increase the reproducibility of findings through the provision of open science neuroimaging data, data management, and data processing algorithms (Hayashi et al., 2024). The continuously growing platform provides simplified access to data from multiple sources and open projects (the OpenNeuro.org, Nathan-Kline Institute, and Human Connectome Project, among others), including data for visualizing brain anatomy and function. Brainlife provides extensive documentation regarding its use via tutorials, videos, and public lectures (on YouTube). This material forms the core educational material to be exported as a CURE suitable for students with little or no previous neuroscience background.

*Course-based Undergraduate Research Experience (CURE)*. Course-based Undergraduate Research Experiences (CUREs) integrate authentic research into regular class activities (Dolan, 2016), addressing questions relevant to the broader scientific community (Auchincloss et al., 2014). This model enhances scalability, provides earlier exposure to research, and promotes inclusivity by allowing more students to participate without the barriers of traditional individualized research experiences. Lawrence Technological University’s College of Arts and Sciences began implementing Course-based Research Experiences (CRE) in 2014, transforming traditional courses into research-oriented learning environments across various disciplines, including biology, chemistry, physics, and psychology. This initiative has successfully revamped over 40 courses with the involvement of more than 30 instructors (Delogu et al., 2023, Shamir et al., 2019). The goals of LTU’s CRE program align with national efforts to improve student persistence in STEM fields and make research accessible to diverse populations. By conducting research during regular class hours (see for example Delogu et al., 2022), CUREs are particularly beneficial for students with non-academic responsibilities, like working students or students with childcare. LTU’s approach stands out due to its scale and multidisciplinary focus, aiming to reshape the pedagogical vision of the entire college. This initiative not only enhances students’ academic experiences but also prepares them for future educational and career opportunities in research-related fields. Previous efforts have successfully applied the CURE model to undergraduate neuroscience education. The Journal of Undergraduate Neuroscience Education (JUNE) has played a key role in showcasing innovative strategies for integrating research into neuroscience curricula and in providing a platform for undergraduate researchers. For example, Ryan and Casimo (2021) described a CURE in which students progressed from formulating research questions to presenting findings using the Allen Brain Map. Similarly, Maldonado-Vlaar and García-Arrarás (2024) outlined effective strategies for expanding research access at Hispanic-Serving Institutions through targeted training programs.

Building upon these foundational efforts, our study introduces a novel integration of cloud computing into a neuroscience CURE framework. By leveraging accessible cloud-based tools for computational neuroscience, our approach offers a new scaffolding that supports scalability, inclusivity, and diversity in neuroscience education—especially for students who might otherwise lack access to advanced research experiences.

In this study, we summarize a pedagogical experience of four cohorts of undergraduate students at Lawrence Technological University. In four consequent semesters, the students of behavioral neuroscience experienced undergraduate course-based research in computational neuroscience by using the apps, the datasets and the cloud computing capabilities of the brainlife platform. This initiative specifically targeted students who lacked prior knowledge or experience in both cloud computing and computational neuroscience. The initial pilot experience, conducted in 2021 focused on examining the accessibility and effectiveness of the cloud computing platform environment when paired with the hands-on collaborative undergraduate research (CURE) approach. The objective was to assess how user-friendly and engaging this combination could be for novices, providing insights into its potential as an educational tool for fostering foundational skills in these emerging interdisciplinary fields. Specifically, we intended to investigate if 1. Students with limited or no previous experience with neuroscience and cloud computing could successfully perform anatomical and functional brain imaging analysis in a cloud computing environment; 2. The course-based experience with computational neuroscience is formative and engaging. 3. Our CURE pedagogical experience is highly flexible and can be used to answer always new original questions. A total of 42 students participated in the CURE experience with brainlife in 4 subsequent semesters (11 in 2021, 11 in spring 2022, 13 in spring 2023 and 7 in spring 2024).

The first semester CURE course (2021) was focused on a general exploration of the platform, the apps and the datasets, while the successive three cohorts of students (2022, 2023, and 2024) focused on specific volumetric hypotheses. As follows, we will describe the work of the first cohort in detail.

*First iteration of the CURE brainlife experience at LTU*.

## Methods

### Participants

All students enrolled in the spring 2021 Behavioral Neuroscience course at Lawrence Technological University (*N* = 11, 6 female) participated in the research activities. The students had no previous experience with Brainlife and no, or very limited, knowledge of brain anatomy and functions.

### Apparatus: combining CURE with a cloud computing neuroscience platform

We combined a CURE pedagogical framework with the use of brainlife.io to structure the learning experience of the students. Specifically, the course was delivered online in a synchronous modality in which students met twice a week for 1 h and 15 min. Most of the assignments required the students to run applications (akin to MRI data analysis steps) on BrainLife and to present the results within a week. The CRE activities were integrated in the course and spanned for the entire 16 weeks of duration of the course.

### Summary of the activities

The course included traditional lectures about theory of neuroscience and practical experiences. The lectures included introductory notions of history of neuroscience, anatomy and physiology of the nervous system, macro anatomical and functional organization of the human brain, brain lateralization, introduction to brain imaging and MRI and fMRI analysis (see Appendix A for a list of materials and readings).

The practical experience included course-based research activities. All CURE activities were conducted in brainlife and the results of the assignments were stored within the platform. The experience was divided into two parts (1) Tutorials and (2) Group analysis through pipeline creation.

#### Part one–Tutorials

Brainlife provided students with tutorials to allow them to familiarize with apps and processes in computational neuroscience. Typically, the completion of each one of the tutorials took a week (two class periods). For each tutorial, the instructor introduced the topic during the class period (on zoom), clarified the meaning of new terminology and concepts and guided the students in their first steps within the tutorial and to plan the activities of the assignment. Our CRE student sample spent time working individually on the following tutorials:

- *Introduction:* this tutorial guides students through account and project creation, access and use of datasets, launching processes, visualizing results
- *Anatomy:* It allows students to familiarize with T1-weighted anatomical images of brains and shows how to process anatomical data for further data analysis (ie. volumetric analysis)
- *FMRI preprocessing:* It shows how to successfully process functional data for further data analysis
- *FMRI networks:* It allows students to generate functional connectivity matrices following fMRI preprocessing.

#### Part two–Group analysis through pipeline creation

Using pipelines, which allowed for the combination of application and processes, students applied the algorithms of the apps they learned in the tutorials to multiple subjects at a time. The students worked individually and as a group to create multiple pipelines with the use of apps indicated in Table 1. All Apps are referenced using the digital object identifier (DOI) provided by brainlife.io under the shoulder: https://doi.org/10.25663.

**Table 1:** Applications used to create a data analysis pipeline for brain imaging research.

App name	Function	DOI
HCP ACPC Alignment	Aligns a T1 weighted image using the anatomical landmarks of the anterior and posterior commissure	https://doi.org/10.25663/brainlife.app.800
FreeSurfer	Segments the T1w anatomical data into functionally different parts of the brain.	https://doi.org/10.25663/brainlife.app.462
Multi-Atlas Transfer Tool	Maps the anatomy of a subject’s brain to a template then subdivides the brain into known brain areas.	https://doi.org/10.25663/brainlife.app.470
fMRIPrep	Preprocesses the functional activations (fMRI) to reduce artifacts.	https://doi.org/10.25663/brainlife.app.160
fMRI to Connectivity Matrices	The fMRI to connectivity matrices app builds functional brain networks	https://doi.org/10.25663/brainlife.app.167
Conmat 2 Network	Converts a conmat datatype to a network datatype so it can be used in the network pipeline	https://doi.org/10.25663/brainlife.app.335
Network Visualization	Generates simple 2D static visualizations for networks	https://doi.org/10.25663/brainlife.app.306

### Dataset and data

Anatomical MRI and fMRI data from 24 subjects in the *human hippocampal replay during rest prioritizes weakly learned information and predicts memory performance* dataset by Schapiro et al. (2020) were used in this analysis. Specifically, Avigan et al. (2020), tracked item-level replay in the hippocampus during an awake rest period after a memory test. The dataset includes two sessions per subject for a total of 48 acquisitions. Data was acquired using a 3 T Siemens Skyra scanner. In each session, they collected 9 functional runs with a T2*-weighted gradient-echo EPI sequence (36 oblique axial slices: 3 × 3 mm inplane, 3 mm thickness; TE = 30 ms; TR = 2000 ms; FA = 71°; matrix = 64 × 64). Each run contained 195 volumes. They collected two anatomical runs for registration across subjects to standard space: a coplanar T1-weighted FLASH sequence and a high-resolution 3D T1-weighted MPRAGE sequence. An in-plane magnetic field map image was also acquired to correct for EPI distortion. More information about data acquisition can be found in Avigan et al. (2020).

### CURE work with brainlife

#### Re-Alignment of the brain and artifact correction

**Figure 1:**
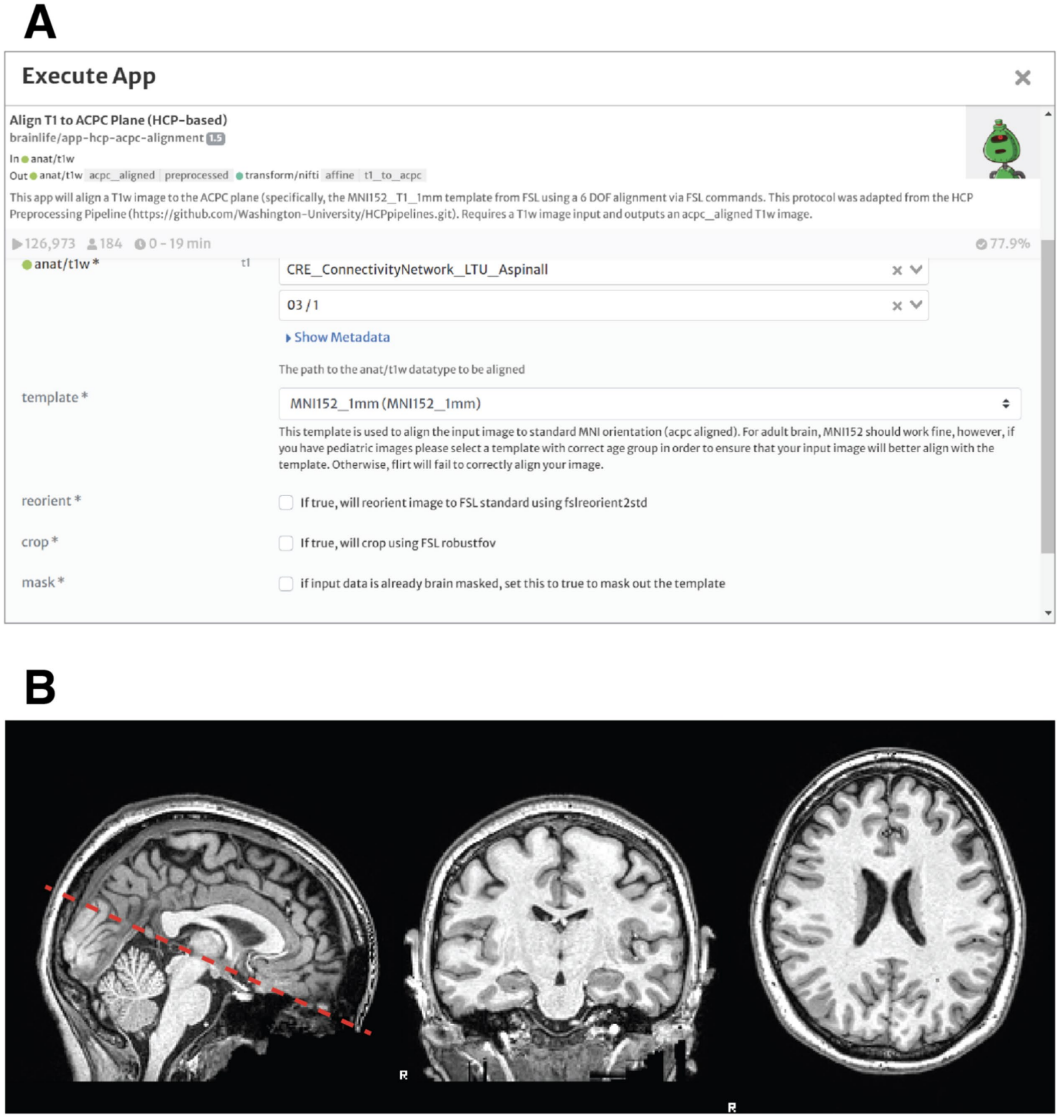
Realignment of the brain and artifact correction of anatomical MRI data. **(A)** Prompt for the execution of the acpc-alignment app in brainlife. The students can select the input file from a list of anatomical t1 weighted files (in this case subject 3, scan 1). The output will be a new anatomical image, realigned to the anterior and posterior commissure (ACPC). **(B)** Sagittal, Coronal and Axial Views of the brain of Subject 3 after ACPC Alignment.

After familiarizing with the brainlife.io environment through the introduction to brainlife tutorial, students performed a standard realignment task to make sure to align the anatomical image to the point where two white matter tracts cross hemispheres: the anterior commissure and posterior commissure (ACPC). Specifically, the image will be moved so that a horizontal line drawn from the front to the back of the brain will pass through both landmarks, and a horizontal line drawn from the temporal lobes and a vertical line from the top of the brain to the bottom bisect at the midpoint of the two landmarks. Typically, this is done by linearly-aligning an anatomical T1w image to a standard template (ex. MNI) that has already been aligned to these planes. The students practiced the alignment of the anatomical image to the anterior and posterior commissure (ACPC) using the Align T1 to ACPC Plane (HCP-based) app. Figure 1 shows an example of the image centered and aligned to the ACPC plane. To properly display the images, another app, Generate Image of T1 was used. The Generate Image of T1 app creates quality assurance images allowing users to view T1 in the mid- axial, sagittal and coronal plane.^2^

#### Measuring the brain: morphometric data extraction and parcellation

**Figure 2:**
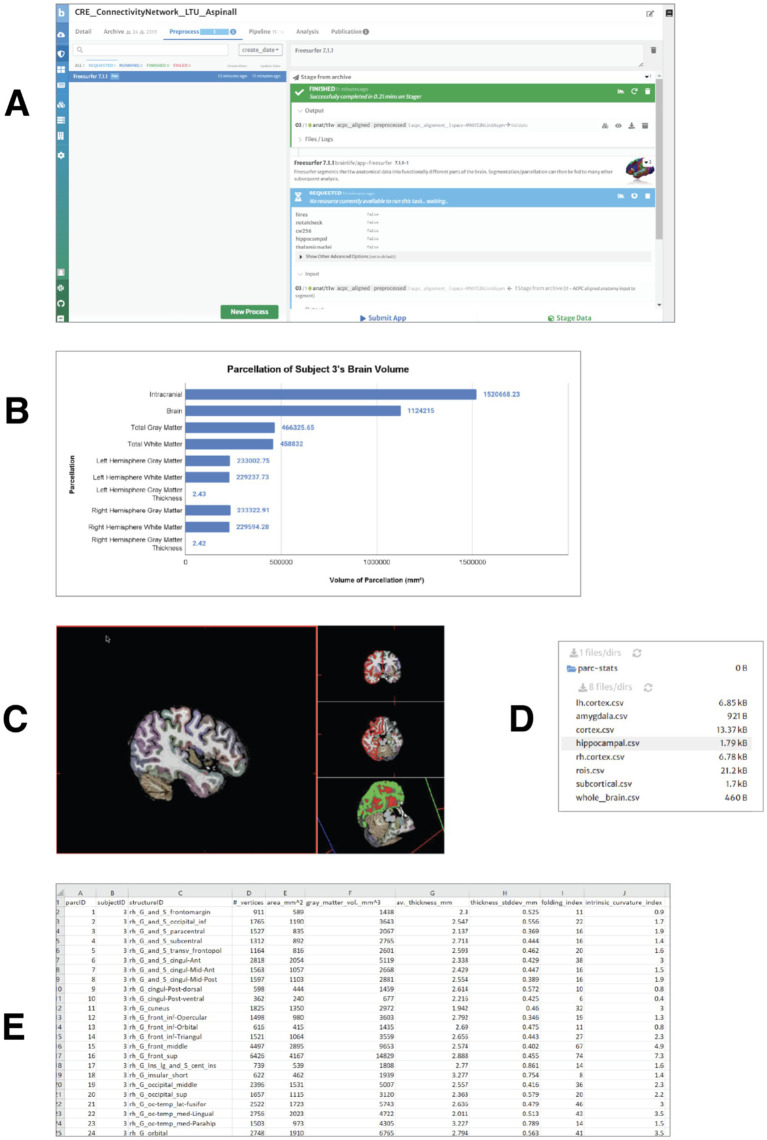
Measuring the brain volumes: morphometric data extraction and parcellation. **(A)** Processing request submitted for the realigned T1-weighted anatomical image of subject 3 using the FreeSurfer 7.1.1 app. The application generates several output files, including a FreeSurfer visualization file for navigation of brain structures and three volume files with brain parcellations based on widely used brain atlases. **(B)** Gray and white matter volumes for subject 3. All measures are reported in cubic millimeters (mm^3^), except for cortical thickness, which is expressed in millimeters (mm). **(C)** Visualization of gray matter, white matter, and cerebrospinal fluid (CSF) volumes in the brain. Distinct cortical structures are represented by different colors. **(D)** Output. csv files generated by the FreeSurfer Statistics app, available for download. These files contain volumetric, surface area, cortical thickness, and vertex count data for cortical and subcortical structures. **(E)** Example of volumetric and morphometric data from the right hemisphere, extracted from the rh.cortex.csv file for subject 3.

The second step for the students was to explore how the whole brain is organized into several functional and anatomical regions. The aligned, anatomical brain image was divided (parcellated) into several anatomical landmarks. The parcellation was achieved through the use of the Freesurfer app (see Figure 2A). As an output of freesurfer, the students obtained a graphical representation of the volumes of gray and white matter and cerebrospinal fluid (CSF) in the brain of one of the subjects (Figure 2B). Volumetric information such as this can be used by students to build and test hypotheses about the relationship between anatomical and functional features of brain areas.

After obtaining general information about the gray and white matter volumes and thickness in the two hemispheres, students produced visualizations in which different cortical structures are represented in different colors and can be explored, navigated in 3d (Figure 2C).

They used the Freesurfer Statistics app^3^ to convert the morphometric information used by freesurfer into tabulated numeric data (comma separated values, csv files) about the volumes for gray and white matter (see Figures 2D,E).

The tabulated data format can be easily used for statistical analysis.

#### fMRIPrep–volume output

**Figure 3:**
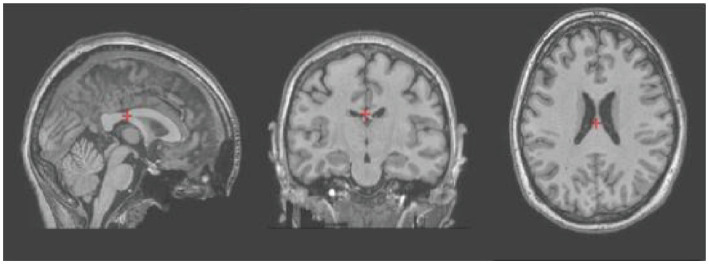
t1-weighted sagittal, coronal and axial images of subject 3 after artifact and distortion correction using the fMRIPrep app.

The next step was for the students to familiarize themselves with preprocess fMRI data via the app fMRIprep. It involved fMRIPrep automatically handling various data corrections and alignments, ensuring the images were ready for their subsequent analytical studies. This step prepared both task-based and resting-state fMRI scans for analysis (Figure 3).

#### Multi-atlas transfer tool

**Figure 4:**
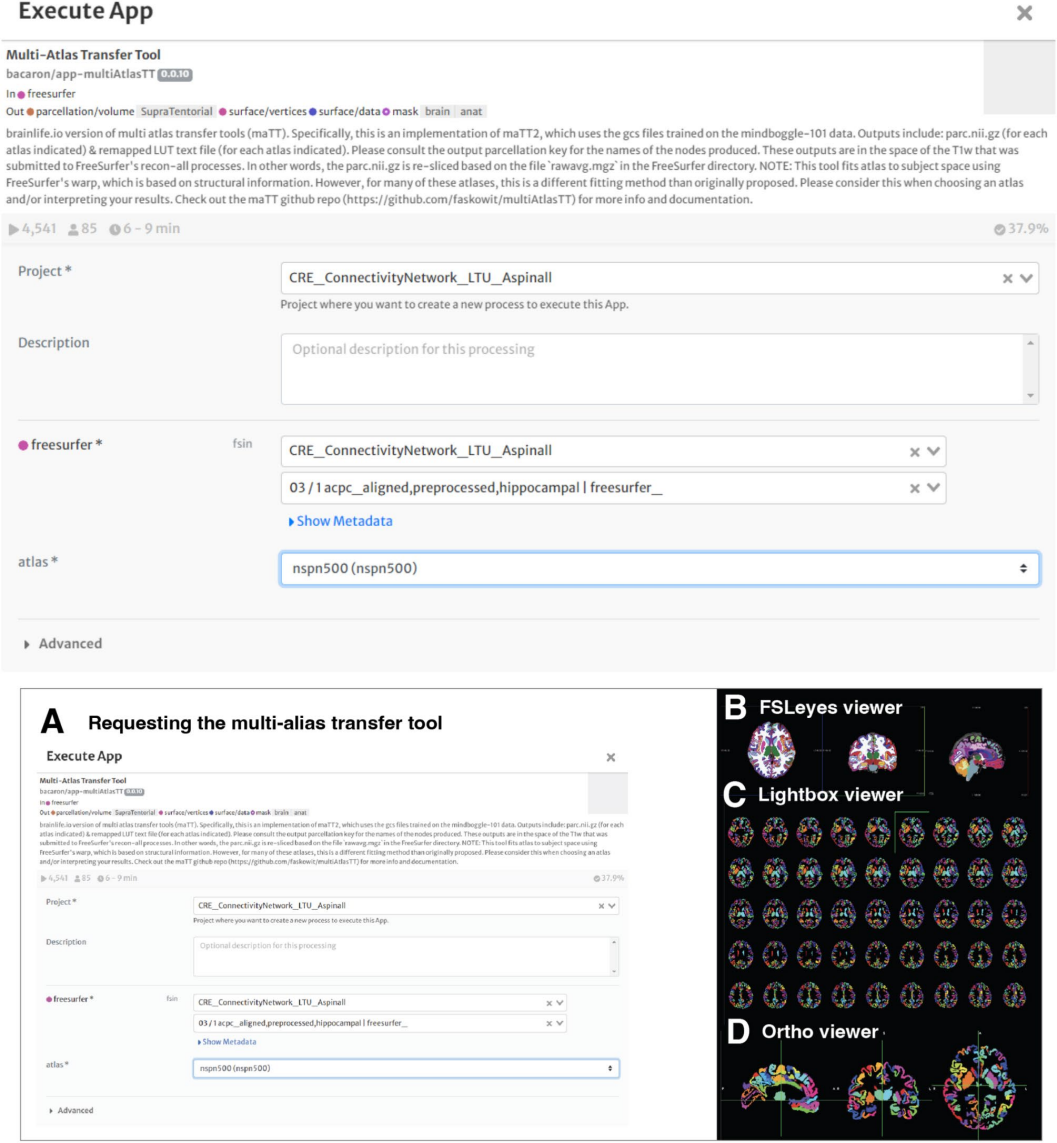
Working with the multi-atlas tool. **(A)** The student requested to process the freesurfer file with the multi-atlas transfer tool map. The app offers flexibility in the volumetric analysis and comparison. **(B)** Brain Parcellation of Subject 3 – Sagittal, Coronal and Axial Views are visualized using FSLeyes - 2D/3D brain volume viewer, which allows visualization of data and results in a variety of useful ways. **(C)** Lightbox view. **(D)** Ortho view.

Completion of artifact reduction and brain parcellation through Freesurfer prepared the images for the use of the Multi-Atlas Transfer Tool application. This application is an important tool to obtain multiple parcellations of the volumetric data within the T1-weighted image (Figure 4).

#### fMRI to connectivity matrices, conmat 2 network and network visualization

After familiarizing with structural MRI by processing anatomical data and images, the students started exploring introductory network neuroscience. Specifically, they focused on network connectivity by using the brainlife apps *fMRI to Connectivity Matrices*, *Conmat 2 Network* and *Network Visualization* measuring and visualizing the correlated activity between different brain regions and got an introductory understanding of how brain regions interact and process information. They first used *fMRI to Connectivity Matrices* which allows identifying functional connections between different regions of the brain by measuring the correlation of activity across multiple regions of the brain, which result in functional connectivity matrices. Between the outputs, there is a comma-separated-values (csv) file containing a bidimensional matrix with all the correlations of activation between brain regions. Such matrices reveal network-level properties of the brain, particularly in fMRI studies. The connectivity of different brain regions are determined by the association of the fMRI BOLD signals originating from the respective areas. Specifically, the connectivity is measured as a correlation between BOLD signals in two or more regions. Functional connectivity matrices have multiple uses, including brain states clustering, characterization of dynamic functional states, identification of individuals, and the understanding of task-related network configurations, all important assets for students learning the foundations of computational cognitive neuroscience.

**Figure 5:**
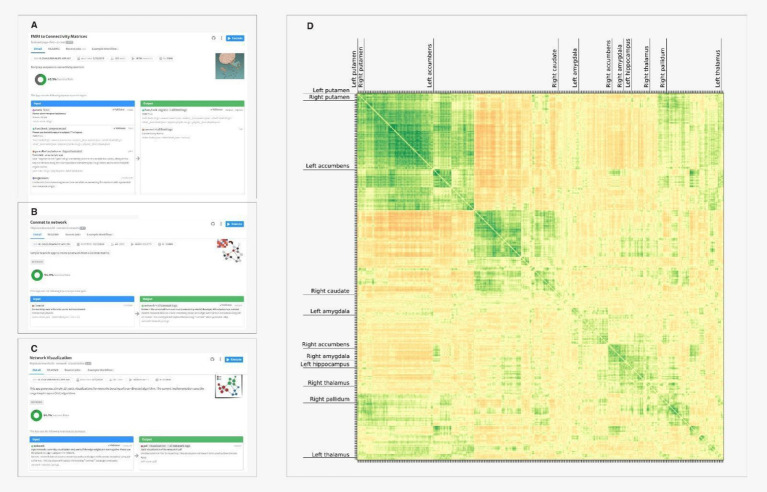
Poster presented by undergraduate students enrolled in a behavioral neuroscience course at LTU research day on April 2022 (https://osf.io/emka8). During spring 2022 semester, all students enrolled in Behavioral Neuroscience explored, through CURE and brainlife, the effects of cocaine user disorder (CUD) on gray matter volumes of parcellated cortical structures. Students extracted volumetry information of 70 brain structures (Desikan-Killiany Atlas) from about 150 brain scans included in 12 different MRI datasets. Results indicated that average volumes of brain structures are consistent between volume data generated from different datasets.

Once functional connectivity matrices were built, the students used the *Conmat 2 Network* app to convert network matrices into network data types that can eventually show the interconnectedness of the different brain regions. With network visualization, the students were able to visualize the network associations from the output of the connectivity matrices. Figure 5 shows the chain of apps used and resulting matrix of correlation between the simultaneous activation of different areas of the brain.

**Figure 6:**
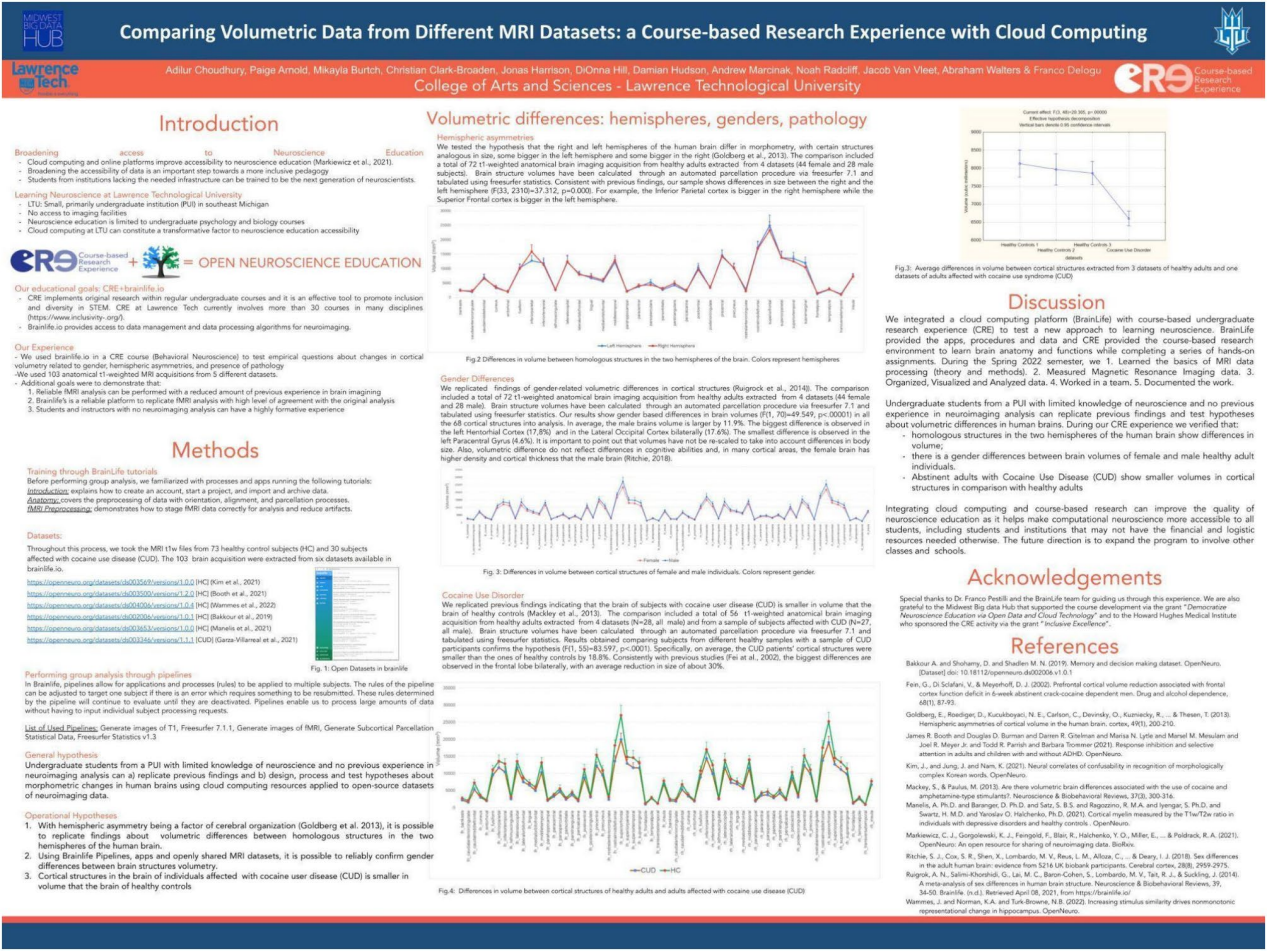
Visualizing the matrix of correlations of activation between regions: **(A)** The student uses the app fMRI to connectivity matrix to obtain a conmat file. **(B)** Conmat 2 Network app is used to convert network matrices into network data types. **(C)** The network visulalization converts network data types in static bidimensional images visualized in pdf outputs **(D)** the correlation matrix visualization. Hue and intensity of colors represent the strength of correlation. Each row and column represent a different brain region. Only few labels were printed for legibility.

### Assessment of the experience of the first cohort of students

Eleven undergraduate students (6 female; 5 male, average age: 20.4) with no previous experience neither with neuroscience analysis nor with any cloud computing platform participated in the first cohort of CURE -brainlife at LTU. The experience was part of a 16-weeks behavioral neuroscience course delivered online. During the course, all students completed the above described assignments in brainlife, studied introductory concepts of neuroscience, neuroanatomy, and neuroimaging. We assessed both performance and experience indicators.

#### Student performance indicators

To assess student performance we took into consideration students’ final grades, the percentage and the timeline of completion of individual assignments and the quality of the CURE final report. The average final grade expressed in numbers was 96.5. A T-Test for single means indicates that the final grade in the CURE course was higher (*t* = 3.68, *p* = 0.001) than the average final grade (92.8) for the same course in 3 previous semesters. The sample used for comparison includes all grades in the non-CURE implementation of the same course in the same university, using the same handbook with the same instructor. This result does not necessarily imply that CURE improved the average grade of students in the course. It could be, for example, that online delivery was an intervening factor. However, the scores in the tests relative to the lecture part of the CURE course were not lower than the scores for the same portion of the curriculum in the traditional implementation of the course. Through the LTU’s learning management system (Canvas), we calculated the percentage of completed tasks. The completion rate of the individual and team tasks was above 80%.

#### Student experience indicators

**Figure 7:**
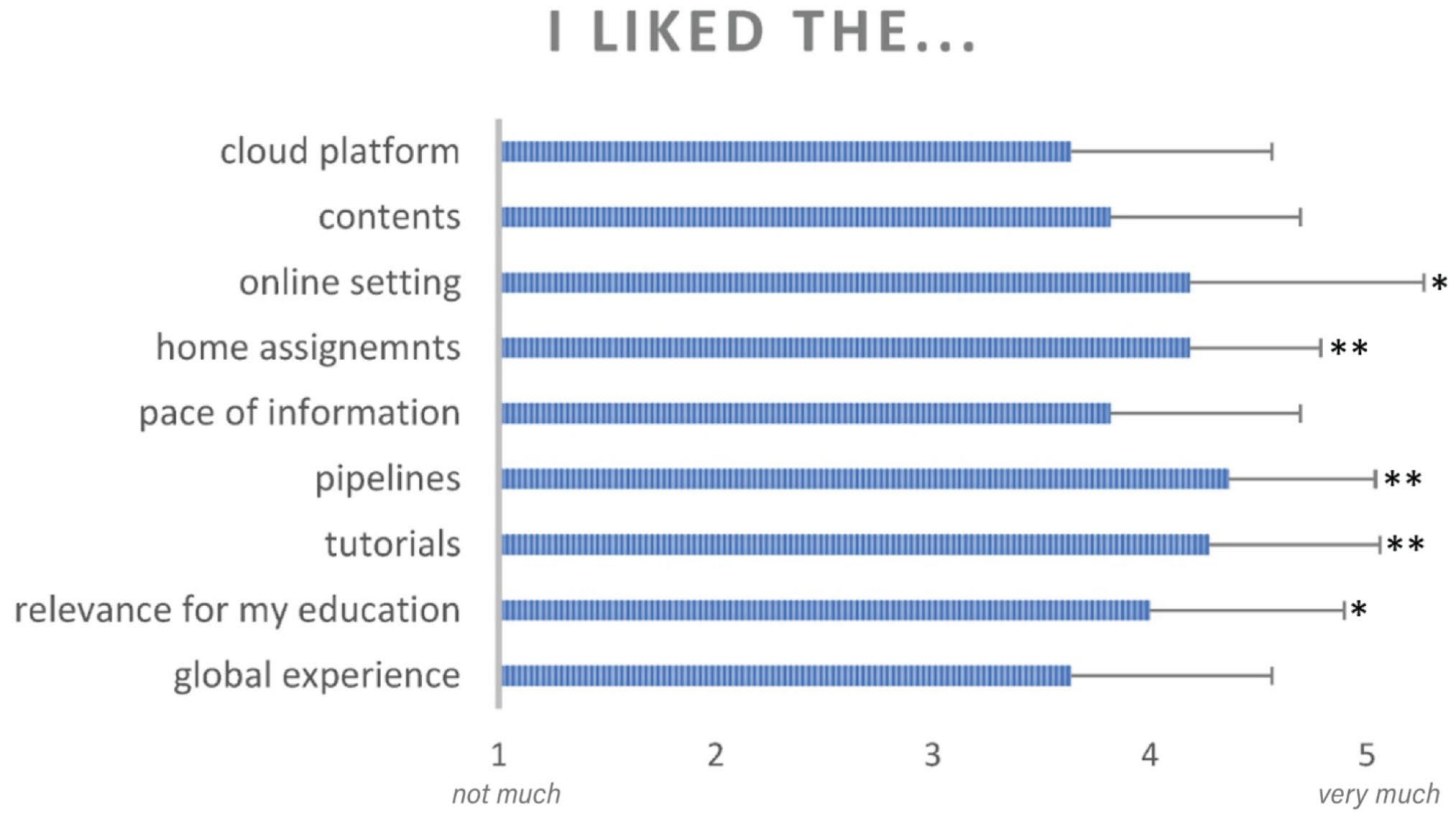
Students reported, in a 5-point likert scale, their perception of the experience with different aspects of their CURE activities and brainlife components. Asterisks indicate significant differences at *p* < 0.05 (*) and *p* < 0.001 (**) from a neutral response. Error bars indicate standard deviation from the mean.

**Figure 8:**
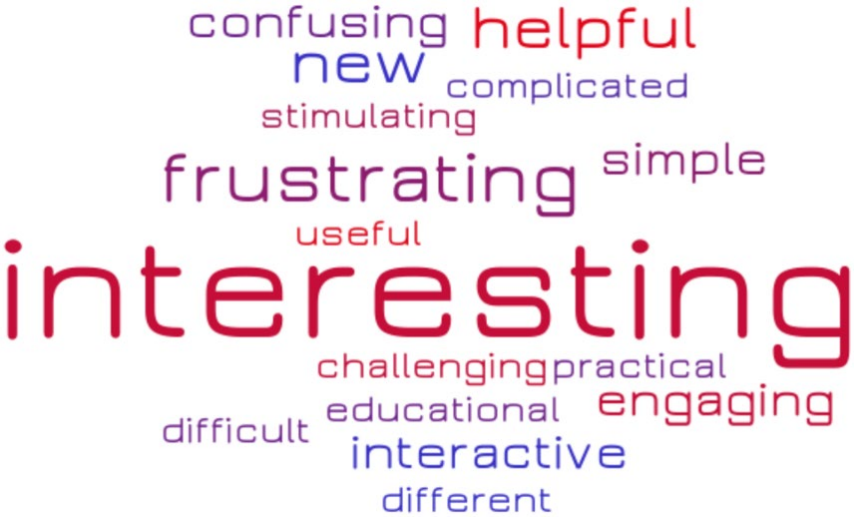
Self reported student experience word cloud. Word size indicates the number of times the word was reported in a list of three adjectives used by the students to describe their CURE experience.

At the end of the course, all students completed a post-course survey indicating, on a scale from 1 to 5 their level of satisfaction with the different aspects of their CURE experience. Results are shown in Figure 7. Each student was also asked to provide a list of two or three adjectives to describe their experience with the brainlife platform, with brain anatomy and with their tasks and assignments. The word cloud shown in Figure 8 indicates the frequency with which every adjective was used. Finally, students were asked to provide a written feedback on the positive and negative aspects of their experience.

As shown in Figure 7, students reported high levels of satisfaction in all aspects of their CURE experience. All average responses were higher than the neutral response value of 3. A single-mean t-test after Bonferroni correction for multiple-testing showed values significantly higher than the neutral response for the following questions: relevance for my education (average response = 4.0, *p* = 0.03), brainlife tutorials (average response = 4.27, *p* = 0.002), completing multi-subject pipelines (average response = 4.36, *p* = 0.0004), and working on home assignments (average response = 4.18, *p* = 0.0006), working in an online setting (average response = 4.18, *p* = 0.04).

The word cloud in Figure 8 shows that “interesting” emerged as the most frequent adjective used to describe the experience. Notably, however, the second most frequent adjective was “frustrating.” Students often reported discussed with the instructor the long processing time of some of brainlife apps as the main cause of frustration during the CURE experience. Other reasons for frustration were the limited time dedicated to theoretical explanations of the brainlife computational processes and of the theory of neuroimaging.

Concerning students’ written feedback on CURE, the qualitative analysis of the written feedback provided by the students indicates a general positive attitude toward the experience. Two independent raters analyzed 9 feedback sentences and rated their valence in a 0–10 scale where 0 is extremely negative, 10 is extremely positive and 5 is neutral. Results show an average valence of 6.1 which indicate a greater focus on the positive aspects of the experience than on the negative ones. The inter-rater correlation, measured with Person’s R coefficient, was *R* = 0.95, indicating high agreement between the two raters.

Both the performance and the experience indicators jointly indicate that a hands-on neuroimaging introduction via the CURE pedagogical model and a cloud computing neuroscience platform can be successfully performed by undergraduate students in spite of a reduced, or absent, previous experience in neuroscience and/or cloud computing. The performance and experience indicators were instrumental for us to refine the learning experience of the successive iterations of the CURE experience in spring 2022, 2023 and 2024.

### Further pilot experiences with CURE in computational neuroscience

The inaugural pilot study in 2021 aimed to evaluate the effectiveness of a Course-based Undergraduate Research Experience (CURE) in computational neuroscience, integrated into an undergraduate neuroscience course for psychology majors (Bucciero et al., 2021). The primary objective of our initial CURE focused was to assess the overall response to the cloud computation environment, associated applications, and assigned tasks of students with no prior background in cloud computing or computational neuroscience.

Following the largely positive outcomes of the pilot, subsequent iterations of the behavioral neuroscience course at Lawrence Technological University (LTU) in spring 2022, 2023, and 2024 have been dedicated to fine-tuning the CURE experience, streamlining computational neuroscience activities, run the activities with a precise theoretical scope in mind. These improvements have shifted the focus toward more direct hypothesis testing, emphasizing authentic research experiences.

As a result, our current CURE model prioritizes hands-on experience in original neuroimaging research. Students are now empowered to run their own research projects by creating an hypothesis, importing actual brain data from open-access datasets, processing the data using the brainlife apps and pipelines, analyzing the data, preparing a final research report and presenting it as a poster in an internal conference (see Figure 6), the LTU research day. This event provides a valuable platform for undergraduate students to showcase their research findings and gain experience in scientific communication.

In each one of the three iterations, the theoretical focus was on structural MRI, and specifically on brain volumetry and morphometry. The choice of working with brain areas and brain volumes is multifolded: 1) For undergraduate students with no previous experience with neuroimaging, the concepts of brain areas, shapes and volumes are easier to grasp than other neuroimaging concepts and measures because of their immediate concreteness. In fact, students immediately understand and appreciate the difference in size and shape of brain structures because they are computed with familiar measures as millimeters (e.g., cortical thickness), squared millimeters (brain areas) and cubic millimeters (brain volumes). 2) Students can easily grasp the conceptual variables and hypothesis that they will operationalize in their projects. Specifically, they easily understand the potential causes of variations of brain morphometry, like the exposome (external exposures, lifestyle, biological exposures, social and economic factors), age, gender, degenerative disorders, brain injuries, strokes, tumors. 3) Increase the sense of research ownership by designing their own hypotheses autonomously was facilitated by seemligly direct relationship between the abovemetnioed factors and brain morphometry.

Each semester was focus on specific empirical questions, agreed with the instructor after an introductory training with the platforms and the apps at the beginning of each semester. The following empirical questions were statistically tested during the different iterations of the course:

1. Spring 2022: Does Cocaine Use Disorder cause reduction in brain white and gray matter?
2. Spring 2023: Does the presence and the type of brain pathology (Schizophrenia, Parkinson’s disease and ADHD) cause morphological changes in brain regions in comparison with healthy controls?
3. Spring 2024: What is the impact of different types of brain tumors and their surgical removal on brain volumetry and spared cognitive abilities?

**Figure 9:**
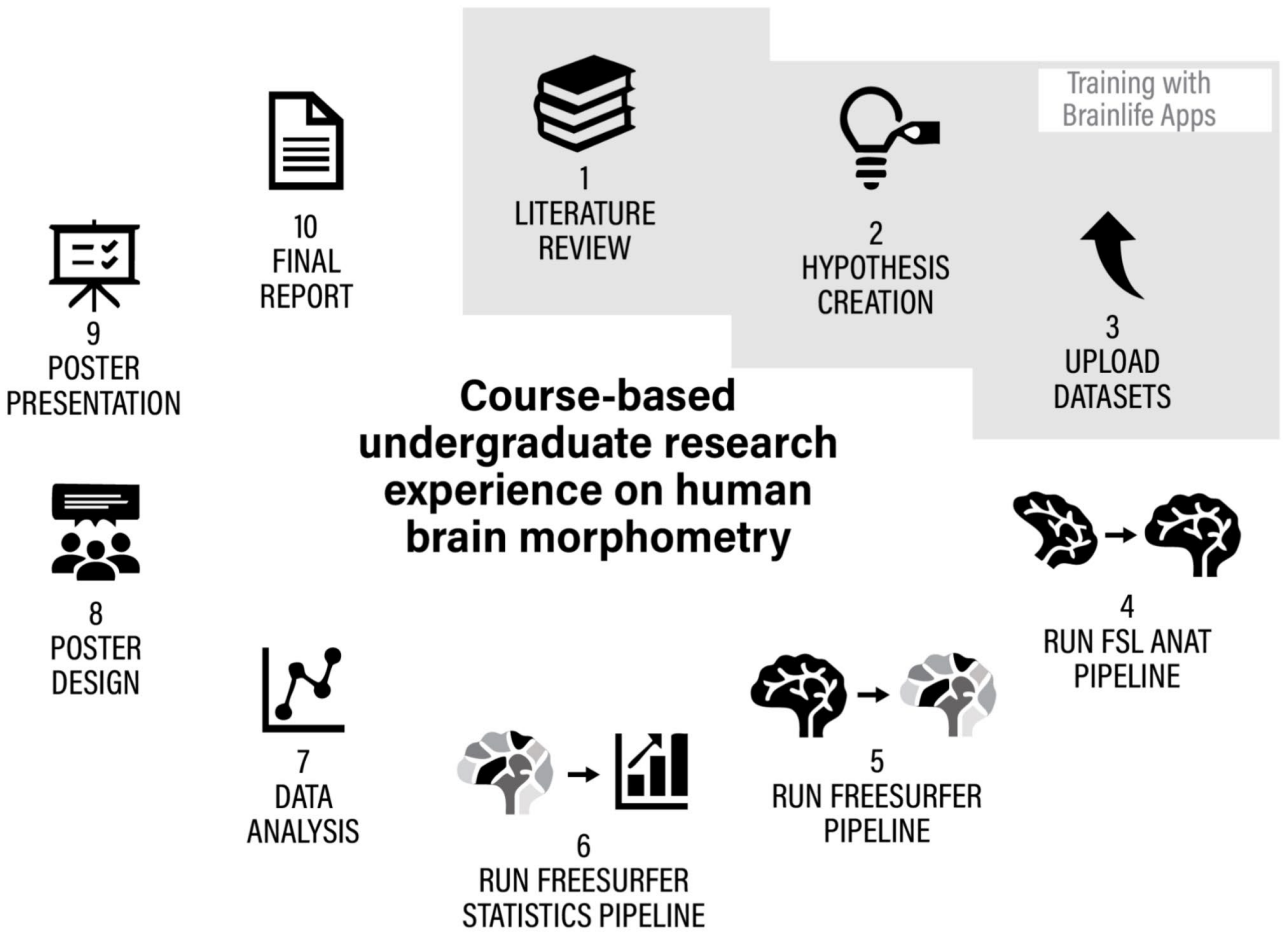
The iterative process of hypothesis creation, testing and reporting of CUREs on structural MRI analysis.

To answer these questions, regardless of the different theoretical focus and empirical hypotheses, the students used very similar processes and procedures, shown in Figure 9 and described in Table 2.

**Table 2:** Description of all the cure tasks, timeline, apps and materials used by students in their CUREs.

CURE task	Task description	Apps	Week	Work type	Materials
1. Literature Review	Explore existing research on factors influencing brain morphometry to identify gaps and formulate objectives.	FSL Anat (single subject)	1–2	individual	Introduction tutorial
2. Hypothesis creation	Develop a testable statement based on literature and project goals.	Freesurfer (single subject)	3	individual	Anatomy tutorial
3. Upload Datasets	Select and upload relevant datasets to the project in brainlife.	Freesurfer statistics (single subject)	4	individual	Openneuro & Brainlife datasets
4. FSL Anat pipeline	Filter, adjust, realign anatomical sMRI data	FSL_Anat (multiples subjects)	5–6	team	Pipeline tutorial
5. Freesurfer pipeline	Extract cortical surfaces and segment brain structures.	Freesurfer (pipeline with multiples subjects)	7–8	team	Pipeline tutorial
6. Freesurfer statistics pipeline	transform the brain parcellations from visualization format to numerical comma-separated-value outputs.	Freesurfer statistics (multiples subjects)	9	team	Pipeline tutorial
7. Data analysis	run statistical analysis, Interpret results, validate findings, and draw meaningful conclusions	Jupyter Notebook, Jasp	10–11	team	Jupyter Notebook
Poster Design	Create a visually engaging summary of findings for presentation.	Apps for image and word editing	12–13	All class	NA
Poster Presentation	Share project insights and results at LTU internal and/or external conferences and workshops.	NA	14	team	NA
Final report	Collective draft of a final report structured like a neuroscience research article. Instructor provide an empty structure, different team focus on one section of the report compiling comprehensive documentation of methods, results, and conclusions.	NA	15–16	All class	Formatted document with step-by-step instructions is provided

The procedures, apps and pipelines described in Table 2 were selected as indispensable, but sufficient tools to test volumetric hypothesis starting just from importing one t1-weighted anatomical file for each subject. Tasks were generally introduced and started during class but mostly completed as home assignments. An exception was the statistical analysis and Jupyter Notebook work, which took place in class under instructor supervision. As coding experience was not required, students with coding skills supported peers with less experience, fostering collaboration and teamwork. Coding sessions followed a guided, peer-supported format rather than formal pair programming. Historically, this process allowed notable discoveries in neuroscience (e.g.: Lawrie and Abukmeil, 1998). Our CURE research protocol and schedule provide students the tools required to run analogous scientific processes to the ones they read in neuroimaging literature already in their undergraduate years. This hyphotesis-driven approach has successfully transformed the course into a more immersive and practical research experience, bridging the gap between theoretical knowledge and real-world neuroscientific investigation. Moreover, most importantly, the protocol allowed students to feel like they own their own discovery process, starting from the hypothesis and ending with a final report ready for student-authored dissemination.

*Spring 2022. The impact of cocaine use disorder on the size of cortical structures.*

All students enrolled in the Behavioral Neuroscience course (*N* = 11) conducted an in-depth analysis of volumetric differences in cortical structures using openly shared MRI datasets. Their investigation focused on three primary hypotheses:

1. The existence of hemispheric asymmetry in cortical volumes.
2. Gender-related differences in brain structure volumes.
3. The impact of cocaine use disorder (CUD) on cortical structure size.

Utilizing Brainlife tutorials and processing pipelines, students analyzed 103 anatomical MRI scans drawn from multiple datasets. Their findings supported existing literature, revealing (a) significant differences in cortical volumes between the left and right hemispheres (see Toga and Thompson, 2003 for a review); (b) Male participants exhibited larger overall brain volumes, while female participants showed greater cortical density in specific regions (see Ruigrok et al., 2014 for a review); (c) Individuals with CUD demonstrated markedly reduced cortical volumes, particularly in the frontal lobe, with an average reduction of 18.8% (see Dang et al., 2022 for a meta-analysis).

Beyond the scientific findings, this iteration of our CURE-brainlife course confirmed that students engaged in authentic research processes can significantly enhance their understanding of course content. Students were particularly impressed to learn about structural changes in the brain correlate with chronic cocaine use. By analyzing real-world data and contributing to hypothesis-driven research, students developed a stronger sense of ownership over their learning. Moreover, working collaboratively on creating the poster and presenting it fostered a sense of community and belonging within the field of neuroscience—factors known to support student retention and success.

*Spring 2023. How age, hemispheric asymmetries and the presence of neurological disorders causes volumetric* var*iations in the brain.*

**Figure 10:**
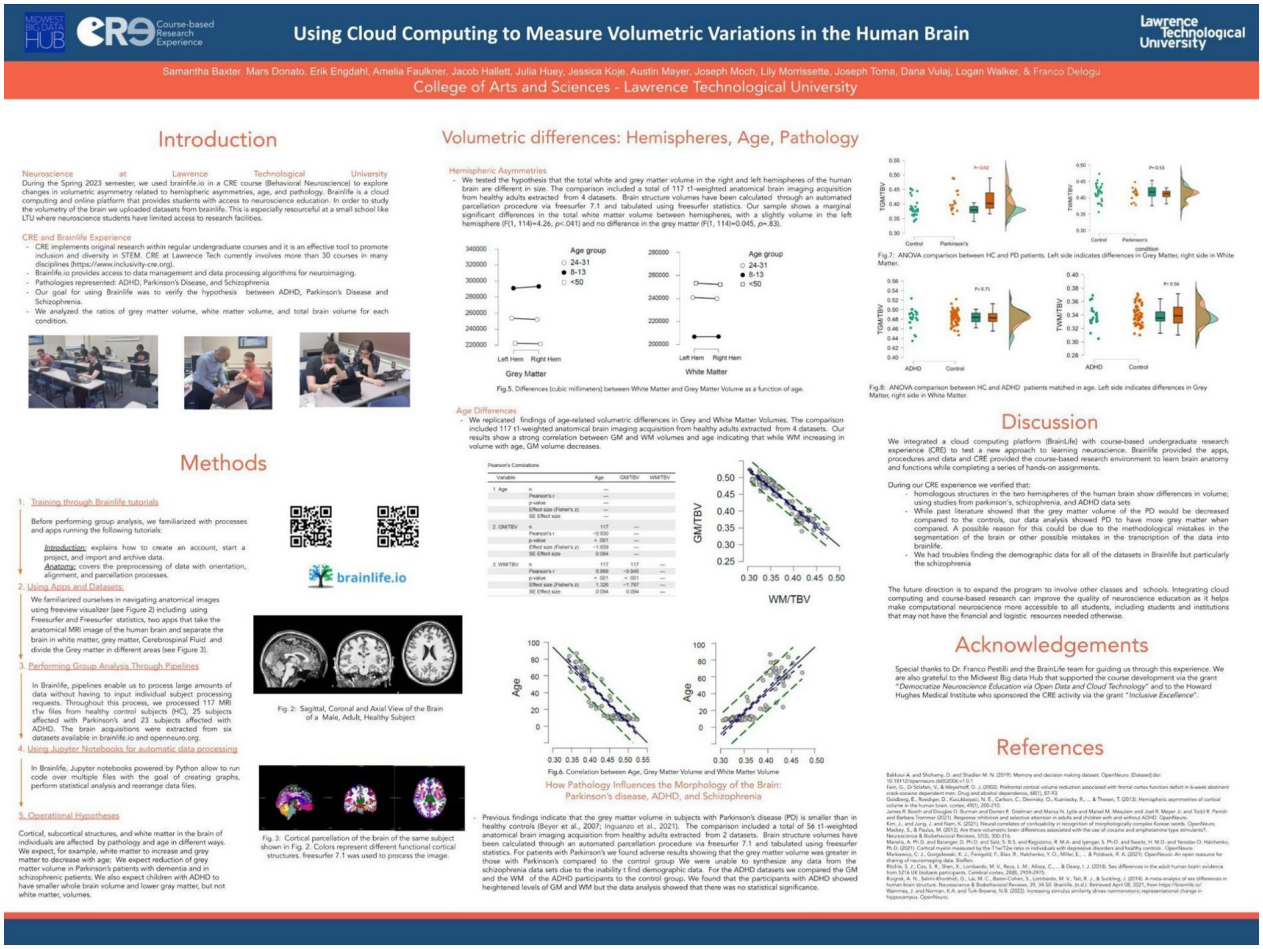
Poster presented by undergraduate students enrolled in a behavioral neuroscience course at LTU research day on April 2023 (https://osf.io/8t3dn). The Spring 2023 class of Behavioral Neuroscience at LTU used brainlife.io to investigate brain volumetric variations (both white and gray matter) as a function of age, gender, hemispheric lateralization and pathological conditions. A total of 200 t1-weighted anatomical MRI scans of brains were selected from 10 different public datasets archived in https://openneuro.org/. The brains were parcellated in 74 non-overlapping areas for each hemisphere using the app freesurfer. Successively, morphometric and volumetric data were reported in csv files using the app freesurfer statistics. The procedure was automated using pipelines to simultaneously run the same apps and procedures over all the subjects included in brainlife projects, individually managed by each student. Finally, a jupyter notebook embedded in brainlife and powered with python was used to perform data tabulation and statistical analysis. Students took into account the following pathologies and related volumetric hypotheses: 1. ADHD, or attention-deficit hyperactivity disorder, with the expectation to find global reductions in gray matter volumes in comparison to control subjects; 2. Parkinson’s, with the expectation to find a reduction in the volume of subcortical areas of Parkinson’s subject compared to controls; 3. Schizophrenia, with the expectation to find areas of the brain cortical and subcortical reduction in schizophrenic patients’ volumes compared to their siblings and to unrelated controls. Consistently with previous studies, students also expected to find a negative correlation between age and gray matter volume as well as gender effects. Results confirmed all the above-mentioned hypotheses.

The spring 2023 semester, all students (*N* = 13) enrolled in Behavioral Neuroscience participated in a course-based research experience. Using brainlife.io, they focused on analyzing volumetric variations in the human brain, emphasizing hemispheric asymmetries, age-related changes, and pathological differences. Like in the previous iterations of the course, our approach allowed students to engage in authentic research despite limited institutional access to neuroscience facilities. The students worked with 117 sMRI brain scans imported from several datasets, including data from healthy adults and individuals diagnosed with Parkinson’s disease (PD), ADHD, and schizophrenia. Guided by brainlife tutorials, they learned to realign and debias sMRI data, perform brain parcellations, and conduct volumetric analyses using tools like FreeSurfer statistics. Key findings included:

- Hemispheric Asymmetry: A slight but significant difference in total white matter volume, favoring the left hemisphere.
- Age-Related Variations: Gray matter volume decreased while white matter volume increased with age, consistent with existing literature (Giorgio et al., 2010).
- Pathological Differences: Unexpected results showed increased gray matter volume in PD patients compared to controls, possibly due to methodological errors or non-homogeneous datasets. ADHD datasets revealed no statistically significant differences between children with ADHD and typically developing children (Figure 10).

*Spring 2024 – investigating the impact of brain tumors and surgical intervention on brain volumetry and cognition.*

During the spring 2024 semester, all students (N = 7) enrolled in the Behavioral Neuroscience course engaged in a course-based undergraduate research experience (CURE), using the BrainLife.io cloud computing platform. Throughout the semester, students investigated the effects of brain tumors and their surgical removal on brain volumetry and cognitive function. This CURE approach, supported by the computational power and accessibility of brainlife.io, enabled students to participate in hands-on, hypothesis-driven research that would typically be out of reach in smaller academic institutions with limited resources.

**Figure 11:**
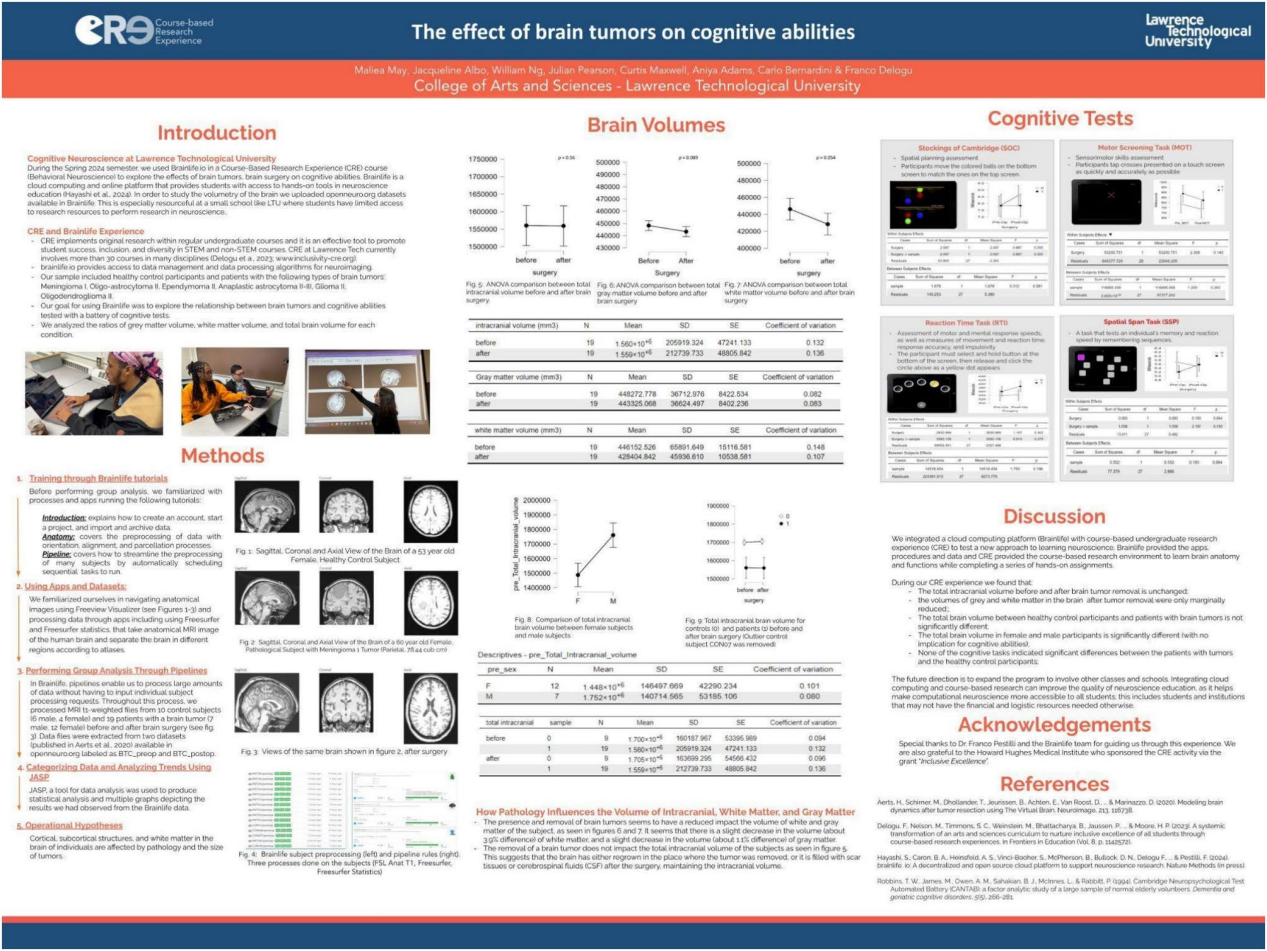
Poster presented at LTU research day on April 2024 about their Spring 2024 experience with CURE-brainlife (https://osf.io/ykbtf). All students enrolled in Behavioral Neuroscience explored, through CURE, the effects of brain tumors, on cognitive abilities. To study the volumetry of the brain, they uploaded datasets available on openneuro.org to brainlife. The sample included healthy control participants (*N* = 10) and patients (*N* = 19) with various types of brain tumors: Meningioma I, Oligo-astrocytoma II, Ependymoma II, Anaplastic astrocytoma II-III, Glioma II, and Oligodendroglioma II. Using the apps available in brainlife, the students analyzed the relationship between brain tumors and cognitive abilities tested with a battery of cognitive tests. They compared pathological (before and after brain surgery) and healthy control subjects in their ratio of gray matter and white matter within the total intracranial volume. They also compared patients and controls for their cognitive abilities. Results indicate that patients with brain tumors did not differ significantly from healthy controls in gray and white matter volumes and in cognitive abilities. The students discussed the evidence that brain tumors, especially when non-cancerous, can be removed with minimal reduction of white and gray matter and that cognitive performance can be successfully preserved.

The research focused on MRI data from 19 patients diagnosed with various types of brain tumors, alongside a control group of 10 healthy individuals. Students conducted volumetric analyses of intracranial space, gray matter, and white matter, comparing pre- and post-surgical scans. Their findings (see poster in Figure 11), revealed that total intracranial volume remained largely stable following tumor resection, potentially indicating compensatory mechanisms such as brain tissue regrowth, scar formation, or cerebrospinal fluid redistribution. Modest reductions were observed in gray and white matter volumes—3.9 and 1.1%, respectively—after surgery. Interestingly, performance on cognitive tasks did not differ significantly between patients and controls, suggesting that brain plasticity may help preserve function despite structural changes.

Through BrainLife tutorials and workflows, students learned key neuroimaging techniques, including MRI segmentation and volumetric analysis, gaining valuable skills in data processing and interpretation. Unlike previous iterations of this course-based research, this project centered specifically on the dynamic changes associated with pathological tissue removal. Students expressed a strong emotional and intellectual engagement with the topic, particularly when visualizing anatomical alterations in tumor-affected brains. This real-world application of neuroscience concepts deepened their understanding of the material and highlighted the relevance of research to human health and clinical practice. In their CURE study, the students did not apply the necessary adaptations to the FreeSurfer pipeline to account for the presence of large brain lesions. This is a crucial consideration, as Freesurfer perform poorly when standard atlas-based is used to analyze structural pathology without corrections. For future iterations of studies involving brain tumors or major brain lesions, it will be essential to implement specialized solutions which can generate a lesion-free T1-weighted image, enabling more reliable and accurate brain parcellation and analysis (Radwan et al., 2021).

Overall, this iteration reinforced the pedagogical value of integrating cloud-based platforms with CURE models in neuroscience education. It not only enhanced technical competencies but also fostered a greater sense of relevance, motivation, and connection between classroom learning and lived experiences.

## General discussion

Over four consecutive spring semesters from 2021 to 2024, undergraduate students at Lawrence Technological University (LTU), a private primarily undergraduate institution (PUI) in Metro Detroit, participated in a computational neuroscience course-based undergraduate research experience (CURE) as part of their Behavioral Neuroscience course. Utilizing brainlife.io, a robust open cloud-computing platform, and students conducted anatomical and functional analyses on brain imaging data sourced from openly available datasets. Each cohort tested original hypotheses about volumetric and structural differences in brain anatomy, reflecting both methodological growth and deepening scientific engagement over time.

The program evolved meaningfully across the 4 years, progressing from a primary emphasis on mastering data processing pipelines and cortical connectivity visualizations to conducting hypothesis-driven investigations on the impact of age, gender, and pathological conditions on volumetric and morphometric properties of the brain. This evolution provided students not only with technical skills in handling and analyzing neuroimaging data but also with an appreciation of the broader scientific questions and clinical implications that guide neuroscience research.

By embedding open science and big data practices in a user-friendly, cloud-based platform, the Brainlife-CURE model offered hands-on, problem-based learning opportunities to students with minimal prior exposure to neuroscience research. BrainLife.io facilitated this by offering unrestricted access to curated neuroimaging datasets, pre-built analysis pipelines, and extensive educational documentation. The platform’s accessibility allowed students to undertake sophisticated neuroimaging research in the absence of high-cost infrastructure or dedicated laboratory facilities—barriers that often limit engagement at PUIs, minority-serving institutions (MSIs), and community colleges.

Crucially, the Brainlife-CURE addressed longstanding limitations associated with traditional undergraduate research models. It demonstrated scalability by integrating research experiences into regular coursework, allowing full-class participation without requiring additional resources or lab space. The approach introduced authentic research early in students’ academic careers, aligning with evidence that early exposure significantly impacts persistence in STEM fields, particularly for underrepresented groups. Moreover, by eliminating selection barriers often associated with competitive research placements, the model promoted greater equity and inclusivity.

Throughout the four-year span, cohorts consistently developed and tested original hypotheses, such as the presence of hemispheric asymmetries, gender differences in cortical volumes, and the effects of substance use or brain pathology on brain structure. In 2024, for instance, students analyzed pre- and post-surgical MRIs of patients with brain tumors, identifying subtle yet informative changes in gray and white matter volumes after tumor resection. In 2023, the class investigated cortical volume variations in individuals with cocaine use disorder, finding significant volume reductions in the frontal lobe—a finding consistent with existing literature. These projects helped student’s bridge classroom learning with real-world applications, including public health relevance and clinical impact.

The program also fostered the development of essential scientific communication skills. Students presented their research at the university’s annual research day, and in some cases, shared their work in peer-reviewed outlets and at professional conferences, such as the Michigan Academy of Science, Arts and Letters. The inaugural 2021 cohort overcame the added challenge of fully remote instruction during the COVID-19 pandemic, highlighting the program’s adaptability to different learning environments.

Overall, this multi-year experience in computational neuroscience demonstrates the feasibility and educational value of a scalable, inclusive CURE model powered by cloud computing. It broadened access to neuroscience education, promoted engagement with real-world research questions, developed technical and scientific literacy, and fostered a sense of scientific identity and belonging among students. By leveraging a wide array of openly available datasets and modular analysis pipelines within brainlife, the program consistently supported new discoveries, making each cohort’s experience unique and impactful.

A key contextual factor contributing to the success of this CURE implementation was the relatively small class size, which enabled close student–instructor interactions and personalized support—especially during coding sessions and while navigating the cloud-based tools. This environment likely fostered higher student engagement and helped to mitigate challenges associated with the technical complexity of the tasks. However, this raises important considerations regarding the scalability of the model. For larger cohorts (e.g., 25–30 students), additional instructional support may be required, such as the involvement of teaching assistants, peer mentors, or the implementation of more structured collaborative learning strategies. Based on our experience, class sizes of up to 20 students can be effectively managed by a single instructor, but exceeding this threshold would likely necessitate adjustments to maintain instructional quality and individualized feedback. Future implementations should explore these adaptations and assess the model’s scalability across diverse institutional settings.

One limitation of the present study is the relatively small sample size and the fact that it was conducted within a single institution, which may limit the generalizability of the findings. However, it is important to note that participating students had no prior experience with cognitive neuroscience, computational neuroscience, or cloud-based scientific platforms. This context renders the sample particularly valuable, as it highlights the potential for successfully introducing advanced, research-oriented content to undergraduate learners without specialized backgrounds. In this sense, the cohort serves as a compelling case study of how open science tools and CURE-based pedagogy can lower entry barriers in neuroscience education.

Another notable limitation relates to the technical infrastructure of the platform. Several brainlife applications experienced instability or long processing times, which occasionally disrupted the workflow. In an educational context, particularly within a course structure that includes assignment deadlines, these issues can be a source of frustration and stress for students—especially those without the technical expertise to troubleshoot such problems independently. This contrasts with the experience of researchers, who may be more equipped to navigate such setbacks. To address this, we recommend continued optimization of the platform’s user interface and backend performance, along with the development of educational-specific features that improve reliability and ease of use. These improvements would significantly enhance the feasibility of integrating brainlife into broader undergraduate curricula.

### Implementation requirements and practical considerations

To support potential adoption of the Brainlife-CURE model at other institutions, especially those with limited resources, we provide below a checklist of the basic requirements, alongside practical insights from our implementation.

Checklist for implementation:

**Table:** 

Category	Requirement	Notes
Hardware	Standard laptops or desktops with stable internet access	No high-performance computing required
Software/Platforms	Free Brainlife.io account	All apps used are freely available within the platform
	Web browser (preferably Chrome or Firefox)	Required to access Brainlife
	Jupyter Notebook (optional, integrated into Brainlife)	For advanced statistical analysis
	Statistical analysis tools (e.g., JASP, Excel)	JASP is free and suitable for beginners
Licensing	None required	All software is open source or hosted in the cloud
Instructor Preparation	Familiarity with Brainlife.io tutorials	Training required for onboarding new instructors
Time Investment	1–2 h per week of tech troubleshooting or support	Depends on class size and instructor experience
Tech Support	Instructor-level support for common issues	No direct institutional IT involvement typically required
Supplemental Materials	Video tutorials, platform documentation	Freely available on Brainlife and YouTube

### Technical challenges and troubleshooting

One recurring challenge we encountered involved the long processing time for certain Brainlife apps and occasional platform instability. These issues occasionally caused delays in assignment submission and student frustration. While these problems were manageable in small classes through instructor guidance and peer support, larger classes may benefit from:

- Having teaching assistants or peer mentors trained in basic Brainlife troubleshooting
- Scheduling flexibility to accommodate potential delays in data processing
- Institutional support for resolving connectivity or access issues (though this was rarely needed at LTU)

In our experience, communication with Brainlife developers the community forum proved effective in resolving most technical concerns.

### Cost considerations

A key strength of this model is its low financial barrier. Since Brainlife is free to use and no physical lab infrastructure is required, the total cost to the institution is minimal. Most costs relate to instructor preparation time and optional printing of final posters or reports. This low-cost, low-barrier model reinforces the scalability and adaptability of the Brainlife-CURE experience, especially in institutions that aim to increase access to neuroscience research for undergraduate students with limited prior exposure.

## Conclusion

Neuroscience continues to be one of the least inclusive STEM disciplines, with many undergraduate students—especially those from underrepresented backgrounds—facing structural barriers such as limited access to neuroimaging data, prohibitive costs of training, and a shortage of diverse mentors. Open science presents a compelling solution to these challenges by promoting transparency, data accessibility, and collaborative learning. Major initiatives such as OpenNeuro and the Adolescent Brain Cognitive Development (ABCD) study have made valuable strides in data democratization; however, they often cater to already-skilled researchers and do not provide structured pathways for entry-level students. As such, there is an urgent need for scalable, inclusive curricular models that embed open science practices into undergraduate education, particularly at institutions with limited research infrastructure.

Our Brainlife-CURE initiative represents a pioneering effort to meet this need. By integrating brainlife.io into the neuroscience curriculum at LTU, we developed a cost-effective, scalable framework for course-based research that allows all students enrolled in a regular undergraduate course to participate in meaningful, hands-on scientific inquiry. Importantly, this model eliminates the need for costly equipment such as MRI scanners or high-performance computing clusters, thereby extending research opportunities to settings that have historically been excluded from high-level neuroscience training. This includes PUIs, MSIs, and community colleges—institutions that are critical for broadening participation in STEM.

In doing so, our program not only reduced the cost of conducting research for students from diverse backgrounds, but also promoted the reuse of openly shared datasets to increase reproducibility and foster innovation. Students explored real clinical and theoretical questions—ranging from hemispheric asymmetries to the neuroanatomical impact of drug use and brain tumors—and made novel contributions to the interpretation of large-scale datasets. These experiences underscore how structured engagement with open science can support both educational and scientific advancement.

Our findings echo the broader literature on open education, which emphasizes that freely available, remixable, and redistributable resources can help reduce inequality in educational access and outcomes (Blessinger and Bliss, 2016; Wiley et al., 2014). At the same time, our program advances the emerging field of open neuroscience education, which has typically focused on upskilling graduate students and postdoctoral researchers (Markiewicz et al., 2021; Rokem et al., 2021; Milham et al., 2018). To our knowledge, only a few programs, such as that described by Oprisan (2022), have specifically targeted undergraduate learners through structured curricula. The Brainlife-CURE thus fills a critical gap, providing a blueprint for how open science practices can be leveraged to introduce undergraduates to neuroscience in a meaningful, inclusive way.

Beyond its educational benefits, this model has the potential to contribute to the progress of neuroscience as a discipline. By expanding access to neuroimaging datasets and analysis pipelines, the program facilitates the discovery of new insights, encourages collaborative scholarship, and supports independent verification of findings—all of which are essential for improving research transparency and integrity.

In conclusion, the Brainlife-CURE initiative demonstrates how open science and cloud computing can be harnessed to transform undergraduate neuroscience education. By combining the principles of course-based undergraduate research with accessible, scalable digital infrastructure, this model empowers students to conduct authentic research, cultivates scientific curiosity, and broadens access to the neuroscience pipeline. As institutions continue to seek innovative strategies for inclusive STEM education, our experience offers a practical, impactful approach to engaging the next generation of neuroscientists.

## Data Availability

The raw data supporting the conclusions of this article will be made available by the authors, without undue reservation.

## Footnotes

^1^In this context, initiatives such as the Open Brain Consent seek to support the brain imaging community by providing guidance on data sharing practices and tools (Bannier et al., 2021). Crucially, such openness is fundamental to promoting a more inclusive educational model, enabling institutions with limited financial resources to effectively train the next generation of neuroscientists.

^2^Description source of Generate Image T1: https://brainlife.io/app/5e88c72d952fefe0a07abfb6/readme

^3^ https://brainlife.io/app/5e31bea8b81ce26c2f9c9a88
