# Supplementary material for: Breaking barriers: broadening neuroscience education via cloud platforms and course-based undergraduate research
Source: Front Neuroinform. 2025 Jul 16;19:1608900. doi: 10.3389/fninf.2025.1608900 (PMC12307389; doi:10.3389/fninf.2025.1608900)
Supplement: Supplementary file 1 [file Data_Sheet_1.pdf]

Appendix A

CURE Course in Neuroscience – Required Materials and Readings

Primary Textbook:
Gazzaniga, M. S., Ivry, R. B., Mangun, G. R., Bassett, D. S., & Phelps, E. A. (2020). *Cognitive Neuroscience: The Biology of the Mind* (5th ed.). W.W. Norton & Company.

Supplementary Readings:

- Greve, D. N. (2011, May). *An Absolute Beginner's Guide to Surface- and Voxel-Based Morphometric Analysis*. Proceedings of the International Society for Magnetic Resonance in Medicine, 19, 33.

- Glasser, M. F., Coalson, T. S., Robinson, E. C., Hacker, C. D., Harwell, J., Yacoub, E., ... & Van Essen, D. C. (2016). *A multi-modal parcellation of human cerebral cortex*. *Nature*, 536(7615), 171–178.

- Eickhoff, S. B., Yeo, B. T., & Genon, S. (2018). *Imaging-based parcellations of the human brain*. *Nature Reviews Neuroscience*, 19(11), 672–686.

These resources were used to support both theoretical instruction and the practical research experience in the CURE course, providing foundational knowledge in brain structure, neuroimaging methods, and parcellation techniques.
